# Pathways for Understanding Blue Carbon Microbiomes with Amplicon Sequencing

**DOI:** 10.3390/microorganisms10112121

**Published:** 2022-10-26

**Authors:** Valentina Hurtado-McCormick, Stacey M. Trevathan-Tackett, Jennifer L. Bowen, Rod M. Connolly, Carlos M. Duarte, Peter I. Macreadie

**Affiliations:** 1 Centre for Integrative Ecology, School of Life and Environmental Sciences, Deakin University, 221 Burwood Hwy, Burwood, VIC 3125, Australia; 2 Marine Science Center, Northeastern University, 430 Nahant Road, Nahant, MA 01908, USA; 3 Coastal and Marine Research Centre, Australian Rivers Institute, School of Environment and Science, Griffith University, Gold Coast, QLD 42222, Australia; 4 Red Sea Research Center (RSRC) and Computational Bioscience Research Center (CBRC), 4700 King Abdullah University of Science and Technology, Thuwal 23955-6900, Saudi Arabia

**Keywords:** Blue Carbon, seagrass, mangroves, saltmarshes, coastal ecosystems, microbiome, rhizosphere, soil, 16S rRNA, synthesis, nutrient cycling

## Abstract

The capacity of Blue Carbon Ecosystems to act as carbon sinks is strongly influenced by the metabolism of soil-associated microbes, which ultimately determine how much carbon is accumulated or returned to the atmosphere. The rapid evolution of sequencing technologies has facilitated the generation of tremendous amounts of data on what taxa comprise belowground microbial assemblages, largely available as isolated datasets, offering an opportunity for synthesis research that informs progress on understanding Blue Carbon microbiomes. We identified questions that can be addressed with a synthesis approach, including the high variability across datasets, space, and time due to differing sampling techniques, ecosystem or vegetation specificity, and the relationship between microbiome community and edaphic properties, particularly soil carbon. To address these questions, we collated 34 16S rRNA amplicon sequencing datasets, including bulk soil or rhizosphere from seagrass, mangroves, and saltmarshes within publicly available repositories. We identified technical and theoretical challenges that precluded a synthesis of multiple studies with currently available data, and opportunities for addressing the knowledge gaps within Blue Carbon microbial ecology going forward. Here, we provide a standardisation toolbox that supports enacting tasks for the acquisition, management, and integration of Blue Carbon-associated sequencing data and metadata to potentially elucidate novel mechanisms behind Blue Carbon dynamics.

## 1. Introduction—Key Knowledge Gaps Amenable to a Blue Carbon Microbiome Meta-Analysis

Belowground microbiomes within seagrass meadows, mangrove forests, and saltmarshes, or Blue Carbon Ecosystems (BCEs), have been studied with the intention to resolve the role of microbes in carbon cycling in these intense carbon sequestration coastal ecosystems [1,2,3,4]. The function of microbiota in BCEs can be quantified through biogeochemical proxies that measure intermediate or final products of metabolic reactions, such as thymidine and leucine incorporation as a measurement of heterotrophic bacterial production [5], stable isotope tracing and biomarkers as proxies for specific microbial activity and biomass [6], and microsensors to measure light or gas flux rates with high spatial or temporal resolution [7,8]. This functional characterisation of microorganisms associated with BCEs revealed the microbial (bio)degradation of organic matter and how this process is coupled with primary production of BCE plants [9,10,11]. This work also highlighted how coastal marine microbiota facilitate the enzymatic remineralisation of high-molecular-weight organic matter, and thus are the “gatekeepers” of the marine carbon cycle [12], with central roles in key biogeochemical mechanisms controlling the decomposition of Blue Carbon [13] and associated emissions of greenhouse gases. In addition to the biogeochemical and metabolic proxy approaches, next-generation sequencing (NGS) technologies have provided researchers with a variety of tools to also characterise microbial taxa and their putative functions explicitly through amplicon sequencing, shotgun metagenomics, and whole genome sequencing [14,15,16]. The convergence of sequencing and biogeochemistry techniques provides an opportunity for a deeper exploration of the Blue Carbon soil microbiome and the mechanisms regulating microbial access to organic matter [13].

In BCEs, carbon accumulation and preservation in soils result from several processes influenced by multiple factors, including local-scale climatic, edaphic, and biological features that simultaneously act on Blue Carbon microbiome structure and function. For example, rhizosphere dynamics are strongly influenced by plant-microbe associations, and as a result rhizosphere microbiomes are highly distinctive from the surrounding soil [17,18,19,20,21]. Additionally, tidal inundation regimes drive redox chemistry, nutrient availability, and plant diversity, which in turn act as strong forces shaping microbial communities [22,23,24]. Further, these interactions between macro- and microorganisms are modulated by abiotic factors sensitive to climate change, such as temperature, precipitation and radiation [25]. In conjunction with these small-scale dynamics, intrinsic features of the habitats themselves, such as soil composition, sedimentation rates, and the depositional nature of the environments also influence carbon quantity and quality [26]. Furthermore, soil microbes themselves can produce an abundance of stable, chemically diverse organic matter, and their interactions with substrates influence the synthesis of soil constituents that are mineral-stabilised [27].

Teasing out the driving forces of Blue Carbon microbiome structures, their role in global carbon cycling processes, and their interaction with organic carbon and the environment across multiple interacting levels (i.e., compartments, organisms, and habitats) may benefit from standardised methods and experimental designs within a large consortia effort [28,29]. Currently, the methodologies for sampling Blue Carbon do not include advice on microbiome experimental design or theoretical considerations [30]. However, studies combining amplicon sequencing with Blue Carbon methods have already begun the process of understanding connections between Blue Carbon chemistry and microbiology [31,32,33]. Amplicon datasets are typically accessible through data repositories, often associated with frameworks for uploading metadata (e.g., MIxS standard [34]). Therefore, in the current absence of a standard methodology, integrating existing datasets could provide an opportunity to produce novel insights into BCE microbiomes beyond the scope of a single study. Such an approach could allow the examination of a Blue Carbon microbiome from a new lens, with the potential to answer fundamental questions about the biological, climatic, and environmental factors that influence microbiome assembly, dynamics, and putative roles in BCEs.

**Figure 1:**
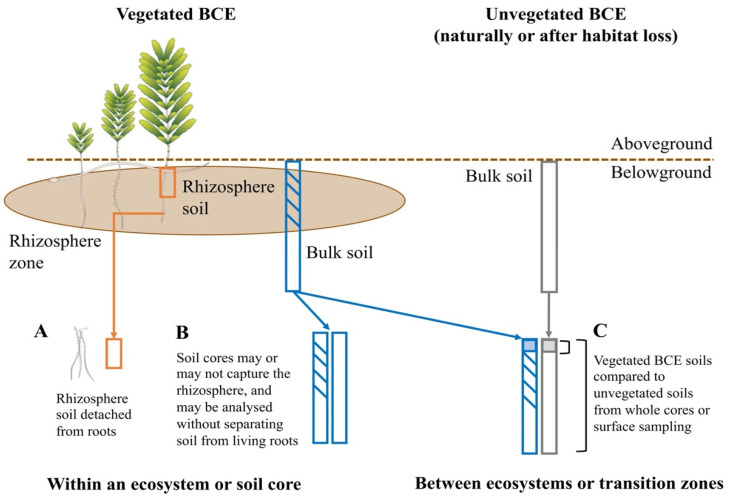
Studying Blue Carbon belowground microbiomes within different compartments based on sampling approaches. Belowground microbial communities associated with Blue Carbon Ecosystems (BCEs) can be studied within a soil core or relatively small scales, e.g., plots (left), or across larger areas, e.g., within or among ecosystems or transition zones (right). Microbial heterogeneity in BCEs is assessed through the comparison of belowground fractions, including the roots and the rhizosphere (**A**), as well as bulk soils (**B**), under the hypothesis that plant-associated interactions play a major role in shaping the microbiome. Vegetated areas (covered by seagrass, mangroves, or saltmarshes) are sampled by coring for microbiome or carbon and may capture partially the rhizosphere (**B**), and are sometimes compared to adjacent, naturally unvegetated areas, such as mud flats and large bare patches, or ecosystems that have experienced habitat loss. Microbiomes within unvegetated BCEs are often compared by sampling bulk soil through scooping (surficial soil) or coring (soil core) (**C**), under the hypothesis that differences in biogeochemistry, redox potential, and food sources for the microbes likely result in different microbial community structure and function, including roles related to carbon and nitrogen cycling. Diagram created using the IAN image library (ian.umces.edu/imagelibrary).

In this study, we first outlined key knowledge gaps on the Blue Carbon microbiomes that can be addressed through synthesis of existing amplicon datasets (Box 1). The list of questions provides examples relevant to working within and between cores, as well as within and across habitat and ecosystem types (Figure 1). We then collated data derived from amplicon (16S rRNA gene) sequencing of microbial assemblages associated with seagrasses, mangroves, and saltmarshes to explore patterns in Blue Carbon microbiomes. More explicitly, we aimed to address each question presented in Box 1, including whether BCEs have a distinct belowground microbiome, inter- and intra-specific and compartment-based variation in their belowground microbiomes, and the possible role of soil carbon density and other environmental forcing in shaping Blue Carbon belowground microbiomes. We considered both the rhizosphere (i.e., microbes closely associated with the roots/rhizomes) and bulk soil microbiome (i.e., microbes associated with bare soils) while reviewing the literature to account for the within- and between-ecosystem compartments at which the Blue Carbon belowground microbiome is studied (Figure 1); namely, relatively small scales within soil cores or plots (Figure 1A,B), and larger scales within or between whole ecosystems or transition zones (Figure 1C). Our approach would advance our understanding of sediment microbiome dynamics in BCEs and generate potentially valuable knowledge for the development of new microbiome methodologies. Moreover, generalised compositional changes (or the absence of them) in Blue Carbon belowground microbiomes could be used to establish microbial baselines to assess disturbance effects and microbial predictors of carbon sequestration and soil health.

Box 1 **Questions that can be addressed through a meta-analysis of BCE amplicon sequencing datasets.**

*Is there a Blue Carbon soil microbiome or a shared “Blue Carbon microbial signature” between BCEs?*

Microbial signatures are extensively supported in the microbiome research literature and have been proposed as predictors of disease occurrence during stressful environmental conditions [35]. Despite the anticipated variability of soil characteristics and microbe–host interactions across samples, there may be a set of shared or “core” microbial taxa or functions amongst BCEs, either in the rhizosphere or bulk soils, that could be used as predictors of carbon preservation or general soil health and function. Identifying a Blue Carbon microbiome signature may also be useful in restoration scenarios, where soils in dysbiosis may necessitate microbiome manipulation strategies to minimise mortality in the face of increasing environmental change [36].

*Is the Blue Carbon microbiome linked to soil carbon content and other Blue Carbon soil metrics?*

Within an ecosystem or soil core (Figure 1A), root system biomass, exudate production, and nutrient exchanges between plants, soil, and microbial communities influence soil carbon and nitrogen concentrations, as well as the microbiome itself. However, how we sample BCE soils to characterise soils, such as organic and inorganic carbon/nitrogen and grain size, would likely affect our interpretations of microbiome datasets within the depth range of the rhizosphere. For example, cores that capture the entire belowground compartment (living roots, rhizomes, and the surrounding soil) would likely produce a different microbial signature than cores from living belowground biomass and bulk soil that were sampled separately. Understanding how these different sampling approaches impact both the microbiology and the chemistry within and across soil cores will be useful for developing standardised microbiome methodologies that add to BCEs soil characterisation.

*What is the effect of environmental and edaphic parameters on the Blue Carbon soil microbiome?*

Carbon cycling in BCEs is the result of several interactions between macro- and microorganisms that are modulated by climate-change-sensitive abiotic factors, such as salinity, temperature, and precipitation [25]. Further, abiotic conditions influence source–sink processes and carbon fluxes between ecosystems or transition zones (Figure 1C) [37,38]. This influence can be either direct or indirect, and relies on complex microbial nutrient transformations; for instance, tidal regimes shaping the microbiome structure and enzyme activity of saltmarshes through redox chemistry and nutrient availability [22,39], or the interaction between exudates and soil metal content shaping the root-associated bacteria of Halophila ovalis [23] (Figure 1A,B). However, the connections and interactions among the microbiome–plant–environment relationship remains a fundamental knowledge gap for coastal microbiomes, with implications on the potential impacts of global environmental change and anthropogenic activities on carbon cycling [29].

*Do inter- and intra-specific variation influence soil microbiomes in BCEs?*

Inter-specific variation accounts for differences between microbiomes that are associated with distinct host species within the same or adjacent ecosystems, including the ecotone. These may or may not share the same niche across horizontal or vertical ranges with variable conditions. In contrast, intra-specific variation involves differences between microbiomes that are associated with different host genotypes or across the biogeographical range of the same species. Both types of variation have been previously reported for seagrass [40,41], mangrove [42,43], and saltmarsh [44,45,46,47] microbiomes, with a focus on each habitat separately. By addressing this question, we may be able to establish generalised patterns of both types of microbial change within and among the three habitats. Understanding the extent to which this variability drives changes in microbial structure in BCEs is essential to disentangle Blue Carbon dynamics; for example, the suppressed decomposition of organic matter [48,49].

*Would this Blue Carbon signature change across different spatio-temporal scales?*

In soils, both deterministic and stochastic processes govern microbial community assembly [50] with strong spatial and/or temporal-scale dependence [51]. In BCEs, factors influencing the rhizosphere within an ecosystem (e.g., root exudation [52]) differ from those affecting the entire habitat and across multiple vegetation types (e.g., tidal frame [53]). Therefore, rhizobiome and bulk soil microbiomes are expected to respond differently and contribute distinctively to the cycling of carbon. Belowground microbiome shifts could also be expected in response to seasonal patterns [54,55] or community successional changes over time [56,57]. Understanding whether and how Blue Carbon signatures change across different spatio-temporal scales is important to identify factors that are critical for carbon preservation and turnover, and to establish microbial baselines against which to measure disturbance effects (e.g., warming and oxygen exposure) and habitat loss that may also impact greenhouse gas emissions [58].

## 2. Materials and Methods—Current Data Availability

**Figure 2:**
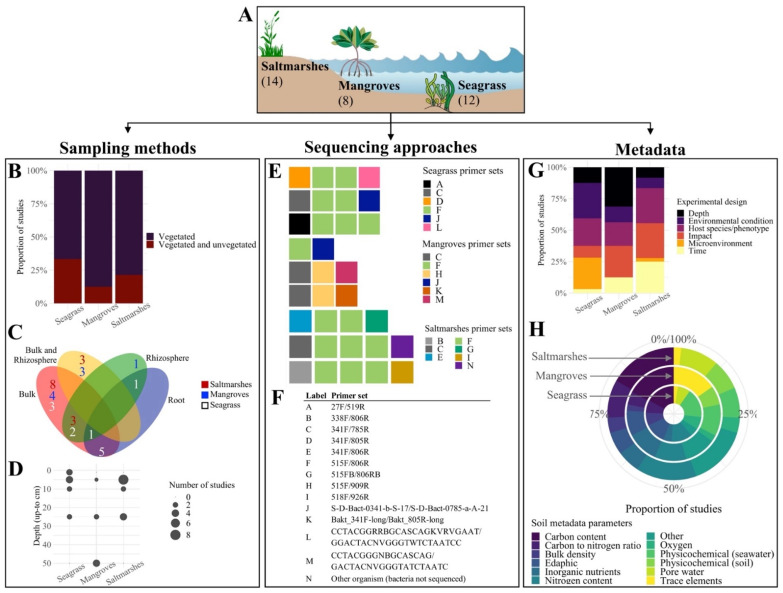
Synthesis of available data associated with Blue Carbon microbiomes. A total of 34 datasets were synthesised from the literature for Blue Carbon Ecosystems (BCEs), including saltmarshes, mangroves, and seagrass (**A**). Our exploration of the data revealed substantial variability across sampling methods, sequencing approaches, and soil metadata. The ecosystems sampled comprised vegetated and/or unvegetated habitats (**B**), while multiple belowground compartments were sampled across BCEs (Bulk = bulk soils, Bulk and Rhizosphere = bulk soils + rhizosphere/roots, Rhizosphere = rhizosphere only, and Root = roots only) (**C**), and a variable depth range from 0 to 50 cm (**D**). Raw 16S rRNA reads were obtained using 13 bacteria-specific primer sets on the Illumina MiSeq or HiSeq sequencing platforms ((**E**), full list of primer sets in (**F**)). Soil metadata were retrieved from 31 studies designed to assess the effects of host species/phenotype, impact, and environmental conditions, among other factors (**G**), and included physicochemical and edaphic parameters, and estimators of carbon and nitrogen content (**H**). Coastline clipart from IAN image library (ian.umces.edu/imagelibrary).

Our synthesis of 16S rRNA amplicon sequencing data associated with Blue Carbon belowground microbiomes involved three stages (detailed methods in the Supplementary Materials). We surveyed the literature through keyword searches in the Google Scholar, Scopus, and Web of Science databases to identify microbiome studies on BCEs. Key-word searches in the Google Scholar, Scopus, and Web of Science databases were performed between June and December 2021, and included the following terms: “16S”, “rRNA”, “microbiome”, “microbial”, “seagrass”, “mangrove”, “saltmarsh”, “salt marsh”, “tidal marsh”, “wetland”, “sediment”, “rhizosphere”, “carbon”, “nutrient”, “Illumina”, and “MiSeq”—see detailed methods in Supplementary Material (File S1 and Figure S1). Illumina sequencing platforms were chosen as selection criteria because it is one of the most commonly used platforms that also has standard methods published for use in environmental microbiome work [59]. Raw 16S rRNA sequences and metadata from studies fulfilling the inclusion criteria, including the V3-V4 hypervariable region as the sequencing amplicon and the availability of soil metadata, were retrieved for further analyses. A total of 34 datasets spanning seagrass (12), mangrove (8), and saltmarsh (14) habitats from 21 countries around the world were compiled (Figure 2A, File S2). Within these, we assessed studies on BCEs with both vegetated and unvegetated (8) and vegetated habitats only (26) (Figure 2B). Although sometimes arguable due to the absence of method description, sample types included bulk soils (sometimes with the intention to capture the rhizosphere), the rhizosphere, and the root system (Figure 2C). Bulk soils were sampled within a variable depth range, including surficial sediments (0–1 cm) and soil cores collected down to 50 cm depth (Figure 2D). Most of the studies used surficial soil sampling at up to 5 cm depth (13), and compared to seagrass and saltmarshes, mangrove soils were up to 10 times deeper. All studies were sequenced through the MiSeq (32) or HiSeq (2) platforms using a variety of 13 primer sets to amplify the V3-V4 hypervariable region of the 16S rRNA gene (Figure 2E,F), with pronounced predominance of the 515F/806R primer set, also used in the Earth Microbiome Project [59]. Additional primers and approaches were used in 18 of the studies to target archaea, microalgae, and fungal taxa. In these studies, more targeted molecular biology techniques (e.g., microarrays, QPCR, TRFLP) were combined with sequencing microbial profiling to assess the response of specific nitrogen cycling genes to environmental change, or to compare distribution patterns and interaction networks between different components of the microbiome (i.e., microbial taxa). These multi-taxa studies were predominantly conducted on mangroves (75%), followed by saltmarshes (50%) and seagrass (42%).

We observed high variability of experimental designs across the 34 datasets compiled. These assessed the effect of wide-ranging environmental or host-associated factors, here grouped into six different categories (Figure 2G). “Host species or phenotype” was the most commonly assessed factor (20 studies), followed by “impact” (17 studies) and “environmental condition” (14 studies). The latter included local environmental descriptors at relatively small (e.g., locations within a seagrass meadow) and large (e.g., the species range in the Northern Hemisphere) geographical scales. Time-series studies were predominantly conducted on saltmarshes (75%), whereas most environmental conditions were evaluated in seagrass habitats (64%).

We were able to retrieve soil metadata from 31 studies (91%), including 10 from seagrasses, 8 from mangroves, and 13 from saltmarshes (Figure 2H and File S2). Soil biogeochemical descriptors ranged from physicochemical parameters (e.g., pH, salinity, and conductivity) measured both in seawater and soil, and edaphic parameters (e.g., dry bulk density, grain size, and water content), to estimators of carbon and nitrogen content (in both organic and inorganic forms), trace elements (e.g., chromium, iron, and zinc), inorganic nutrients (e.g., nitrate, nitrite, sulphate, and phosphate), and pore water profiles. Moreover, oxygen levels and carbon-to-nitrogen ratios were recorded for 19% and 32% of the 31 studies, respectively. Additional metadata included carbon and nitrogen isotopes, redox potential, and indicators of bacterial metabolic rates. The most commonly recorded metadata were carbon and nitrogen content metrics (in 24 and 18 studies, respectively), followed by physicochemical parameters (16 studies). A geographic restriction of carbon inventories has been previously reported in some of these ecosystems [60] and may explain the lack of availability of carbon or nitrogen metrics for some of the studies.

As part of our in silico analyses, we attempted to trim all reads to the same length (positions 515F to 785R of the 16S rRNA gene, detailed methods in the Supplementary Materials). Standardising the region of the 16S with our trimming strategy was necessary to avoid potential bias introduced due to differential primer affinities (Figure 2E,F) that may ultimately lead to biased taxonomic profiles. Our first two trimming tests did not cause major changes in clustering, ordination, or diversity plots. However, differences in read length (i.e., short amplicons not covering the desired region), composition (i.e., absence of primers in the reverse reads), and pre-processing prior to database submission were observed in some of the sequencing data, which prevented the trimming and subsequent pooling of these datasets. Therefore, we analysed each dataset separately—an approach that has been used successfully to find consistent patterns characterising disease-associated microbiome changes across multiple datasets [61]. Lastly, a series of data subsetting steps were performed to assess the suitability of different synthesis approaches (e.g., random forest classifiers) for directly comparable subsets of the collated data (see “Data subsetting” in the Supplementary Materials).

Multiple questions about “universal signatures” of the Blue Carbon belowground microbiome could be answered by the comparison between the observed sample types. However, our first data subset (8 studies with samples from both vegetated and unvegetated habitats) resulted in very low statistical power. The alternative selection of 26 studies with vegetated soils only seemed plausible if pooling of datasets after trimming was possible in this instance. The fact that only 24% of the studies had both vegetated and unvegetated soils may suggests that experimental designs are influenced by within-ecosystem research interests (e.g., nutrient enrichment in saltmarshes and biogeography in seagrasses) or limited funding availability for microbiome research, specifically on BCEs.

The high variability of sampling methods, sequencing approaches, experimental designs, and reporting of metadata resulted in low replication at the study level—despite large samples sizes within studies, the number of studies per habitat type was insufficient for a classification algorithm (such as random forest classifiers) to have the statistical power required to detect an effect. This imbalance in class distribution, where “class” represents each habitat, significantly influences the performance of classifiers, and as a consequence, the resulting statistical power can be dramatically affected [62]. Furthermore, some studies had sampling designs amendable for habitat comparisons but lacked the soil metadata needed to address some of our research questions (Box 1). In conclusion, a robust meta-analysis using currently available BCE datasets was ultimately not possible, because of the low statistical power that would result from the low replication at the study level, and the bioinformatic inconsistencies that prevented us from pooling sequencing reads from different studies.

Notwithstanding, we were able to perform qualitative comparisons between datasets that revealed patterns or features of the BCEs microbiomes. For instance, Proteobacteria and Bacteroidetes were the predominant phyla across habitats, regardless of the specific types of samples collected, the environmental factors influencing the holobiont, and the sequencing approaches used to produce the data. Their prevalence and association with sulphur and nitrogen metabolism suggests that members of these groups could constitute the core BCE microbiome. Firmicutes, Chloroflexi, and Actinobacteria were also dominant phyla within seagrass, mangrove, and saltmarsh ecosystems, respectively. Sequence retrieval from these abundant microbes may be more strongly influenced by primer selection. Moreover, several families within Proteobacteria were reported as differentiating bacteria within below-ground microbiomes associated with BCEs, including Azospirillaceae, Chromatiaceae, Desulfobacteraceae, Hyphomicrobiaceae, Sphingomonadaceae, Vibrionaceae, Xanthobacteraceae, and Xanthomonadaceae. These taxa showed drastic changes in abundance across treatments or conditions (e.g., latitudinal transplantation, vegetation zones, drought, salinity, and restoration of tidal flooding in saltmarshes), suggesting that microbes within these families might be good bioindicator candidates to monitor or predict ecosystem state or environmental change. More generally, below-ground microbial assemblages and their putative functions were more diverse than above-ground microenvironments associated with the holobiont, indicating a higher potential for bioprospecting. Because of the ubiquitousness of aerobic and anaerobic fungi (e.g., invariably found across sediment depths, relative to bacteria and archaea members of the interactome), and adaptability of anaerobic archaea (e.g., predominant taxa in strongly reducing and sulfidic conditions), these microorganisms may be keystone species for carbon storage.

## 3. Results—Opportunities for Blue Carbon Microbiomes through a Standardisation Toolbox

**Figure 3:**
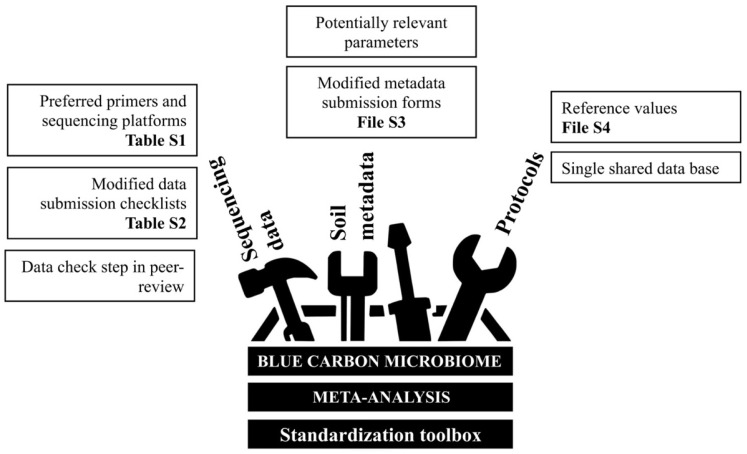
Proposed enacting tasks toolbox. General approaches have been previously suggested to solve methodological barriers to conduct a synthesis or meta-analysis on existing data. Here, we propose ways to improve them using more tangible resources that support enacting tasks. Tasks are clustered into three categories, each responding to the methodological constrains listed in Table 1, and could be actioned in any order. Standardisation tools supporting each task are provided in the Supplementary Materials.

Despite the limitations we encountered in trialing a meta-analysis, our exploration of existing data found recurring themes across microbiome studies in BCEs and revealed barriers in the methodological approaches and data needed to address key research questions in the field (Box 1). The methodological constraints discussed here apply to synthesising existing data (i.e., testing hypothesis with data on hand), rather than to carrying out new research (i.e., conducting experiments in the field or the laboratory). With each methodological constraint, however, we see opportunities for solutions, as well as research avenues that would progress these knowledge gaps (Table 1). Below, we explore the technicalities through existing solutions while also proposing new approaches via a standardised toolbox (Figure 3). With the toolbox, our aim is to provide methodologies and identify potential standards to be adopted by the Blue Carbon microbiome community. This would ultimately allow for a consistent study of Blue Carbon belowground microbiomes and the re-use of research outputs in future evidence-based, comparative investigations such as meta-analyses.

### 3.1. Sequencing Data

Methodological biases of next-generation sequencing technologies represent risks associated with direct comparison of combined data that was generated using different strategies (i.e., data pooling). To avoid primer bias, we searched for studies targeting the V3-V4 region, which was chosen because it has been used predominantly in global microbiome research initiatives aiming to cover close to the entire diversity of a natural microbial community [59]. We observed major inconsistencies across primer sets (Figure 2E,F), in addition to inaccessible or unavailable mapping files to match with raw sequencing files. Formal sample indexes were often rare, and sample information published in the manuscripts was sometimes insufficient to match with amplicon samples. Moreover, some studies had sequencing data in a format not optimal for reuse through the DADA2 pipeline (e.g., reads processed by the sequencing provider, joined paired-reads), and in some cases there were samples with no sequencing files stored in public data repositories. Standardisation tools for the acquisition of amplicon sequencing data proposed here include: (i) a selection of amplicon sequencing primers and platforms for data collection based on the methods reported in the 34 studies compiled here and their use in global microbiome initiatives, (ii) modifications to existing checklists for data submission to reflect the resolution of microbiome sampling (e.g., paired microbiome and soil carbon sampling) or at minimum site-level characterisation at the time of microbiome sampling, and (iii) addition of a verification step to the manuscript production process to assure compliance with standards for reporting marker gene sequences (see “Standardisation toolbox–Sequencing data” in the Supplementary Materials).

### 3.2. Soil Metadata and Experimental Designs

Belowground carbon assessments for BCEs come from biogeochemical and ecological studies with highly variable methodologies that were developed to answer a range of research questions. Fest et al. [67] found multiple inconsistencies in methods currently implemented to estimate carbon stocks in mangroves. While a Blue Carbon Manual exists [30], the authors highlighted that carbon standards must be applied globally to achieve accurate carbon estimations [67]. Percent of soil organic carbon was the most commonly reported parameter (Figure 2G), although how it was described, processed, or reported differed among studies. This simple and commonly reported carbon metric and its normalised density metric (using dry bulk density) are ideal for a microbiome meta-analysis due to its simplicity and commonality for soil studies in BCEs. Carbon mass levels could be therefore predicted through meta-analysis by classifying samples into categories (e.g., low, medium, or high), provided a single soil biogeochemical parameter was measured in all samples. Including these parameters as a required variable on the sequencing checklist (Table S2) would be necessary, as raw values within manuscripts were sometimes only presented in graphical summaries or means and not always available upon request. Standardisation tools for the acquisition of Blue-Carbon-related metadata proposed here include: (i) a selection of sediment biogeochemical parameters to be recorded that are potentially relevant to investigate the Blue Carbon belowground microbiome, (ii) modifications to existing metadata submission forms that would act as the equivalent of standards for reporting marker gene sequences, and (iii) a collaborative approach to agree on standard methods for data collection, archival, and sharing.

### 3.3. Protocols

The Mangrove Microbiome Initiative (MMI) was recently launched as a call for a multidisciplinary and collaborative approach to understand the role of microbes in mangrove productivity and carbon budgets [28]. The authors stressed the need to prioritise three research areas related to microbial characterisation across different scales, the biogeochemical basis of ecosystem functioning, and a holistic view of the mangrove microbiome, while suggesting approaches to advance the field. Among these, standard methods that allow for collaborative studies, controlled settings in reproducible systems, and manipulative experiments. The International Blue Carbon Initiative is governed by the same principles and has published standard methods to produce robust Blue Carbon data [30]. A parallel standard protocol for the identification and characterisation of the Blue Carbon microbiome is currently lacking, although it has been highlighted as a key need to define its composition and function [29]. In this study, we focus on the details required to re-interrogate existing data, based on improvements and recommendations to approaches previously proposed as part of global initiatives, such as the Earth Microbiome Project (EMP, https://earthmicrobiome.org/), the Human Microbiome Project (HMP, https://hmpdacc.org/), the Australian Microbiome Initiative (AusMic, https://www.australianmicrobiome.com/), and the Biomes of Australian Soil Environments Project (BASE, https://bioplatforms.com/projects/soil-biodiversity/). We hope that the toolbox provided in this paper (Figure 3) provides tangible resources that would help inform and guide Blue Carbon microbiome methodologies, so that meta-analysis and other syntheses approaches using existing sequencing data (File S1) can be used collectively in the future.

## 4. Discussion—Recommendations and Conclusions

The Blue Carbon belowground microbiome is a complex system whose dynamics are yet to be fully understood—a “black box” that, if deciphered, would broaden our understanding of global biogeochemical cycles and other ecosystem services provided by BCEs. Indeed, opening the black box of the roles of microbes in carbon cycling would provide an opportunity to render carbon processing actionable, broadening our current slate of actions, which focus on conserving and restoring vegetation, directly and indirectly, thereby conserving and enhancing Blue Carbon stocks and sequestration. Belowground microbiomes associated with BCEs have been studied separately through amplicon sequencing approaches, delivering theoretically suitable datasets for meta-analysis. This is a very powerful predictive tool, extremely valuable for synthesis research and hypothesis generation. However, many of the existing local datasets are difficult to access, subject to license restrictions, or are designed in a way to address questions that sit outside what is needed for a meta-analysis. The current lack of coordinated data infrastructure for Blue Carbon microbiomes within the repositories presents both challenges and opportunities for future development.

Here, we suggest enacting tasks to solve issues that preclude meta-analysis on existing data for the exploration of the Blue Carbon belowground microbiome. Our toolbox is focused on amplicon sequencing approaches to interrogate microbial communities associated with BCEs and their putative functions. However, these tools could be applied to data generated through other sequencing approaches, provided different adjustments specific to the data type are made. For instance, modifications to standards for data reporting, similar to the ones suggested here for marker gene sequences, would facilitate the standardisation of the archiving process for further data re-use regardless of the data type, but the specific items and definitions in the corresponding checklist(s) would differ according to the sequencing platform; e.g., to account for assembly method and sequencing depth in shotgun metagenomics [68]. Moreover, our toolbox could also be applied to standardise protocols other than carbon methods, again with a series of adjustments that satisfy the collection of other potentially relevant parameters that might also influence the Blue Carbon microbiome and carbon budgets, such as redox potential, nutrient or pollutant levels and net primary productivity and biomass of the local vegetation. In some cases, it may also be appropriate to consider water quality parameters that may influence belowground processes, e.g., hypoxic or eutrophic conditions. For this to happen, different biogeochemical parameters would need to be chosen as representative metrics, and different modifications would need to be implemented to standardised sequencing checklists and metadata submission forms. Nevertheless, we hope the tools proposed here can help move the field towards an all-encompassing standardisation. This perspectives paper presents: (i) a summary of key knowledge gaps amenable to meta-analysis on Blue Carbon belowground microbiome data, (ii) an evidence-based foundation for methodological barriers that prevent meta-analysis on existing Blue Carbon belowground microbiome data, and (iii) a standardisation toolbox that supports enacting tasks for the acquisition and management of Blue-Carbon-associated sequencing data and metadata suitable for meta-analysis. Solving these issues would expand our knowledge on BCEs from the still-limited microbiome descriptions that are currently available.

## Figures and Tables

**Table 1:** **Barriers to meta-analyses on currently available data and proposed solutions.** Challenges revealed by attempted exploration of the Blue Carbon belowground or soil microbiome through meta-analysis on existing data, previously proposed solutions, and new suggested approaches.

Research Question	Meta-Analysis Approach	Methodological Constraints	Result	Technical Issues	Previously Proposed Solutions	Additional Solutions (This Study)	Supplementary Materials
Is there a Blue Carbon soil microbiome or a shared “Blue Carbon microbial signature” between BCEs?	Combine multiple studies from seagrass, mangroves, and saltmarshes	Variable sequencing approaches used to generate data from BCEs	Prevents comparisons between datasets through data pooling	Inconsistent primer sets	MIMARKS [34]	Preferred primer sets and sequencing platforms	Primers and sequencing platforms list (Table S1)
				Sample index or mapping file accessibility	Established minimal requirements for SRA/EBI-ENA submissions or alike	Modified submission checklists, including mandatory tabs for data format and sample ID. Example with MIMARKS [34]	Checklist modifications (Table S2)
				Not-optimal sequencing data formats			
				Missing sequencing files or samples in mapping files submitted to data repositories	Implemented data curation in peer-review	Data check step in peer-review; i.e., production editor to assure the submission of complete datasets to the repositories	Not applicable
Is the Blue Carbon microbiome linked to soil carbon content and other Blue Carbon soil metrics?	Run separate random forest classifiers within studies that measure soil carbon density	Normalised carbon density data often not measured	Limited normalised carbon density data, which require measurements of both percent of organic carbon and dry bulk density	Carbon density data not collected	Research focus on resolving finer cause-–effect and correlative details surrounding the microbiome	Latest advances on the topic, with suggested potentially relevant parameters	Not applicable
				Raw data not available (only graphical summaries/averages published)	Global database with parameters set up; i.e., targeted carbon data repositories [63,64]	Modified metadata submission form with mandatory fields. Example with EDI [64]	Form modifications (File S3)
						Invitation for everyone to contribute collaboratively, marketing campaigns	Not applicable
What is the effect of other environmental and edaphic parameters on the Blue Carbon microbiome?	Include multiple environmental parameters as factors	Varying soil metadata, often specific to the treatments or hypotheses of each study	Variable parameters for soil metadata, measured at differing depths	Multiple parameters to inform on carbon content	Suggested standards from global initiatives; e.g., EMP [59], BASE [65].	Suggested reference values. Example using Blue Carbon Manual worksheet [30]	Reference values worksheet (File S4)
				Multiple units for the same parameter		Proposal of a single shared database for established standard methods, protocols, and reference values	Not applicable
Do inter- and intra-specific variation influence soil microbiomes in BCEs?	Example with vegetation type: run random forest classifiers within studies that predict habitat	Few studies with required experimental design; i.e., with vegetated and unvegetated samples collected at the same depth	Reduced statistical power of classification algorithms	Experimental designs influenced by within-ecosystem research interests	Design and implementation of studies to understand influence of vegetation and cross-habitat subsidies of carbon on microbiomes and soil parameters [66]	Not applicable	Not applicable
Would this Blue Carbon signature change across different spatio-temporal scales?	Include studies from different biogeographical locations and seasons	Current microbiome studies influenced by research interests or funds availability	Lack of studies with experimental designs aligned with the key research questions of this study	Not applicable	Not applicable	Not applicable	Not applicable

## Data Availability

The data analysed in this study are openly available. Please see details in File S2.

## Supplementary Materials

The following supporting information can be downloaded at: https://www.mdpi.com/article/10.3390/microorganisms10112121/s1, Figure S1: Study selection (PRISMA 2020 flow diagram); Figure S2: Trimming in silico analysis (515F-806R dataset to 520F-785R length); Figure S3: Trimming in silico analysis (341F-785R dataset to 515F-785R length); Table S1: Preferred sequencing approach; Table S2: Modified MIMARKS checklist; File S1: PRISMA 2020 checklist; File S2: Collated data; File S3: Suggested additional section for Blue Carbon metadata recording; File S4: Suggested reference values for data collection. The following references are only cited in the Supplementary Materials [69,70,71,72,73,74,75,76,77,78,79,80,81].
