# Supplementary material for: Pathways for Understanding Blue Carbon Microbiomes with Amplicon Sequencing
Source: Microorganisms. 2022 Oct 26;10(11):2121. doi: 10.3390/microorganisms10112121 (PMC9694552; doi:10.3390/microorganisms10112121)
Supplement: Supplementary file 1 [file microorganisms-10-02121-s001.zip › microorganisms-1921124_revised-sup-material_clean_proofread/1_microorganisms-1921124_revised-sup-material_clean_proofread.pdf]

Pathways for understanding blue carbon microbiomes with amplicon sequencing

Valentina Hurtado-McCormick[1*#], Stacey M. Trevathan-Tackett[1*], Jennifer L. Bowen[2], Rod M. Connolly[3], Carlos M. Duarte[4], Peter I. Macreadie[1]

[1] Centre for Integrative Ecology, School of Life and Environmental Sciences, Deakin University, 221 Burwood Hwy, Burwood, VIC 3125, Australia; v.hurtadomccormick@deakin.edu.au, s.trevathantackett@deakin.edu.au, p.macreadie@deakin.edu.au
[2] Marine Science Center, Northeastern University, 360 Huntington Ave, Boston, MA 02115, United States; je.bowen@northeastern.edu
[3] Coastal and Marine Research Centre, Australian Rivers Institute, School of Environment and Science, Griffith University, Gold Coast, QLD 42222, Australia; r.connolly@griffith.edu.au
[4] Red Sea Research Center (RSRC) and Computational Bioscience Research Center (CBRC), 4700 King Abdullah University of Science and Technology, Thuwal 23955-6900, Kingdom of Saudi Arabia.; carlos.duarte@kaust.edu.sa
[*] Authors contributed equally.
[#] Correspondence: v.hurtadomccormick@deakin.edu.au

Supplementary information file

Table of contents

- **Detailed methods:** Data collection, Preliminary *in-silico* analyses, Data subsetting…….…………..p. 1-2
  - **File S1**: PRISMA checklist …………………….………….....Additional supplementary .docx file
  - **Figure S1**: PRISMA flow chart…………………….…………….…………………………….………..p. 3
  - **File S2**: Collated data from literature search….……………Additional supplementary .xlsx file
  - **Figure S2**: Trimming strategy, trial 1…………………………………...……..…………………..p. 4
  - **Figure S3**: Trimming strategy, trial 2………………………………………………………………..p. 5
- **Standardisation Toolbox**: Sequencing data, Soil metadata & experimental designs, Protocols......p. 6-8
  - **Table S1**: Preferred amplicon sequencing platforms and primer sets…………………...…....p. 9
  - **Table S2**: Modified MIMARKS checklist…………………………………………….…..…….p. 10-11
  - **File S3**: Modified metadata submission form……………..Additional supplementary .docx file
  - **File S4**: Reference values…………………………........….. Additional supplementary .docx file

1. Detailed methods

Our synthesis of 16S rRNA amplicon sequencing data associated with Blue Carbon soil microbiomes evidenced current gaps that prevent meta-analysis, and hence avert the possibility to direct future experiments and novel hypotheses beyond those of any one individual study. This section outlines how we discovered such gaps, while providing evidence-based foundation for the methodological constraints further elaborated in the manuscript.

1.1 Data collection

We first surveyed the literature to identify microbiome studies on Blue Carbon ecosystems (BCEs). Key-word searches in the Google Scholar, Scopus, and Web of Science databases were performed between June and December 2021, and included the following terms: "16S", "rRNA", "microbiome", "microbial", "seagrass", "mangrove", "saltmarsh", "salt marsh", "tidal marsh", "wetland", "sediment", "rhizosphere", "carbon", "nutrient", "Illumina", and "MiSeq" – see details on Preferred Reporting Items for Systematic Reviews and Meta-Analyses (PRISMA) in **File S1** and **Figure S1**. Additional studies were identified by following references in related microbiome studies on coastal, marine, and estuarine soils not specifically associated with the rhizosphere. We included studies with publicly available raw 16S rRNA data (read files in FASTQ or FASTA formats), sequenced using the Illumina platforms MiSeq or HiSeq (paired-end reads) with primers targeting the V3-V4 hypervariable regions of the 16s rRNA gene (e.g., 515F/806R), and with metadata indicating the type of sample (rhizosphere vs. bulk soil), soil biogeochemistry (e.g., bulk density, grain size, texture, etc.) or carbon/nitrogen content (e.g., total C, % organic C, dissolved organic C, C:N ratio, etc.). These inclusion criteria were chosen based on the recognised higher quality of paired-end MiSeq reads [69], and to make the data as comparable as possible while avoiding bias. Biased amplification likely results from different primer choices, sequencing platforms, or library preparation protocols [69,70], varying polymerase amplification efficiencies [71], and low sequence diversity or unbalanced base composition in template DNA sequences [72]. Sequencing reads and corresponding metadata were downloaded from online repositories (e.g., read files from SRA or ENA, metadata from PANGAEA or EDI) or links provided in the original publications, or were acquired directly from the authors (**File S2**). Once irrelevant publications were excluded, a total of 34 datasets spanning seagrass (12), mangroves (8), and saltmarshes (14) habitats from 21 countries around the world were compiled for a series of subsetting steps subsequently performed to assess different meta-analysis approaches (**Figure 2**).

1.2 Preliminary in-silico analyses

Even though we only selected studies with data from the V3-V4 regions of the 16S rRNA gene, primer sets varied substantially across datasets. A true comparison would have required data amplified with the same primers, which, to the best of our knowledge, did not exist. Therefore, we attempted to trim all reads to the same length (515F-785R) for consistency purposes – i.e., all reads would correspond to the V4 region. Standardizing the region of the 16S rRNA gene with our trimming strategy was necessary to avoid bias introduced due to differential primer affinities that ultimately lead to biased taxonomic profiles. Trimmed sequences would have been used as input for the DADA2 pipeline [73]. We ran a series of preliminary *in-silico* tests to assess if trimming to the 515F-785R length would affect general patterns in the data and thus result in misleading biological interpretations.

First, we manually trimmed a 515F-806R dataset to a 520F-785R length (a few bases from each end), using the *trimLeft = c(5,5)* and *trimRight = c(21,21)* parameters in DADA2. This did not cause major changes in clustering, ordination, or diversity plots (**Figure S2**). Second, we trimmed a 341F-785R dataset to a 515F-785R length, using CUTADAPT [74] as a treatment step before DADA2. Forward reads were trimmed based on the 515F primer sequence, while reverse reads were primer-clipped using the 785R primer sequence. This caused very slight changes in clustering, ordination, or diversity plots (**Figure S3**). The third test was intended to assess if the same would happen when analysing a 341F-785R and a 515F-806R datasets together (both trimmed to a 515F-785R length). Inconsistencies between the reverse reads prevented CUTADAPT to find primers sequences and therefore cut at the 785 position. More specifically, primers sequences were not found

when reads were checked manually. This reflected the overwhelming effect of slight biases in individual primers on the ecological patterns of our data, which were not as robust as we expected. Based on these results, we decided to analyse each dataset separately rather than pooling the data after trimming reads to the same length – an approach that has been successfully used to find consistent patterns characterising disease-associated microbiome changes [61]. Methods such as these must be implemented with caution, as the study outputs cannot be directly compared (i.e., the data cannot be merged into a single taxonomic table for downstream analysis), and only patterns within studies should be used for results interpretation.

1.3 Data subsetting

There was a lot of variability across our 34 16S amplicon sequencing datasets, mostly from differences in the sample types collected (bulk soil vs. rhizosphere) and the available soil biogeochemical data. Experimental designs were also highly variable across studies, making our datasets not directly comparable. To address this issue and to investigate the potential existence of "universal" microbial signatures of vegetation in BCEs (rather than variable microbial structure shaped by vegetation type), we first focused on experimental designs with both unvegetated (i.e., bare) and vegetated (i.e., adjacent to the roots) soils. We selected 8 out of the 34 studies (24%) initially compiled for our first meta-analysis, including 4, 1, and 3 studies from seagrasses, mangroves, and saltmarshes, respectively (**Figure 2** and **File S2**). Random forest classifiers are learning algorithms with excellent performance, currently considered one of the strongest models for handling large and noisy datasets [75]. Random forest classification with 16S rRNA gene amplicons have been proposed as a rapid, sensitive, and accurate solution for identifying host microbial signatures [76], and hence seemed like the best method for this dataset. Although sample sizes within each study were large enough to achieve high statistical power, the number of studies per habitat type was insufficient. Moreover, samples collected by Garcias-Bonet et al. (2020) did not have associated soil biogeochemical data. Excluding this study would have reduced even further the statistical power of our meta-analysis. Indeed, lack of consistent associated metadata was a recurring issue. When reported, differences between parameters also challenged the comparability of the data, and therefore subsetting studies by those with a minimal set of shared soil biogeochemical metadata (e.g., 7, 8, and 9 studies reporting carbon content in seagrass, mangroves, and saltmarshes habitats, respectively; **Figure 2**) was not an option either. The alternative was to subset the data to include only studies with vegetated soils, irrespective of biogeochemical data availability. This approach would have resulted in a selection of 26 datasets (76%), including 8, 7, and 11 studies from seagrasses, mangroves, and saltmarshes, respectively (**Figure 2** and **File S2**). Although these studies had large sample sizes and were apparently comparable, these data would have not been suitable for meta-analysis because multiple studies could not be combined into a single working dataset due to the issues evidenced by our preliminary *in-silico* analyses.

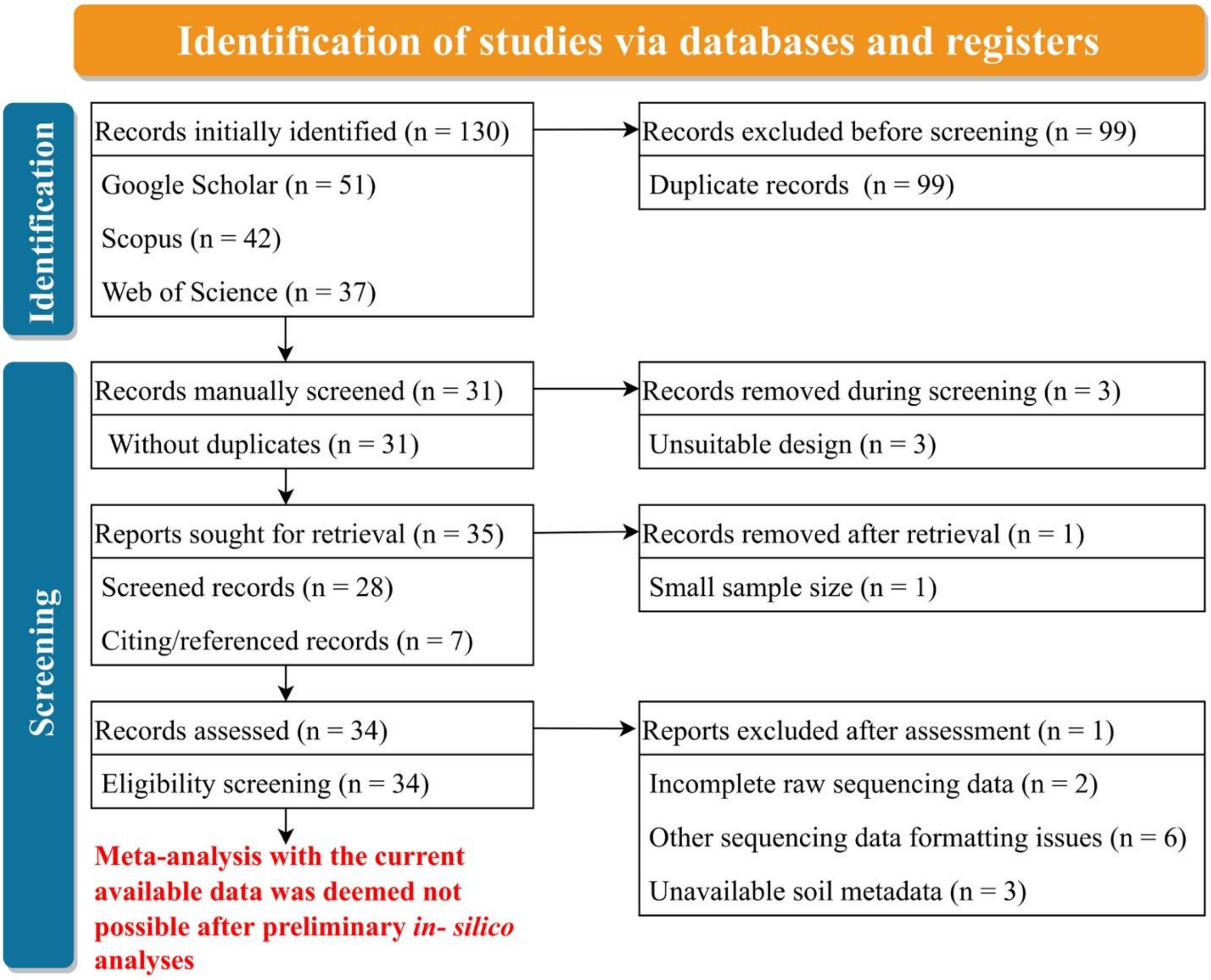

Figure S1. Study selection (PRISMA 2020 flow diagram). Results of the search and selection process, from the number of records initially identified in the search to the number of studies excluded before the meta-analysis was declared unsuitable. PRISMA 2020 flow diagram for new systematic reviews for searches of databases and registers only. Adapted from: http://www.prisma-statement.org/ [77].

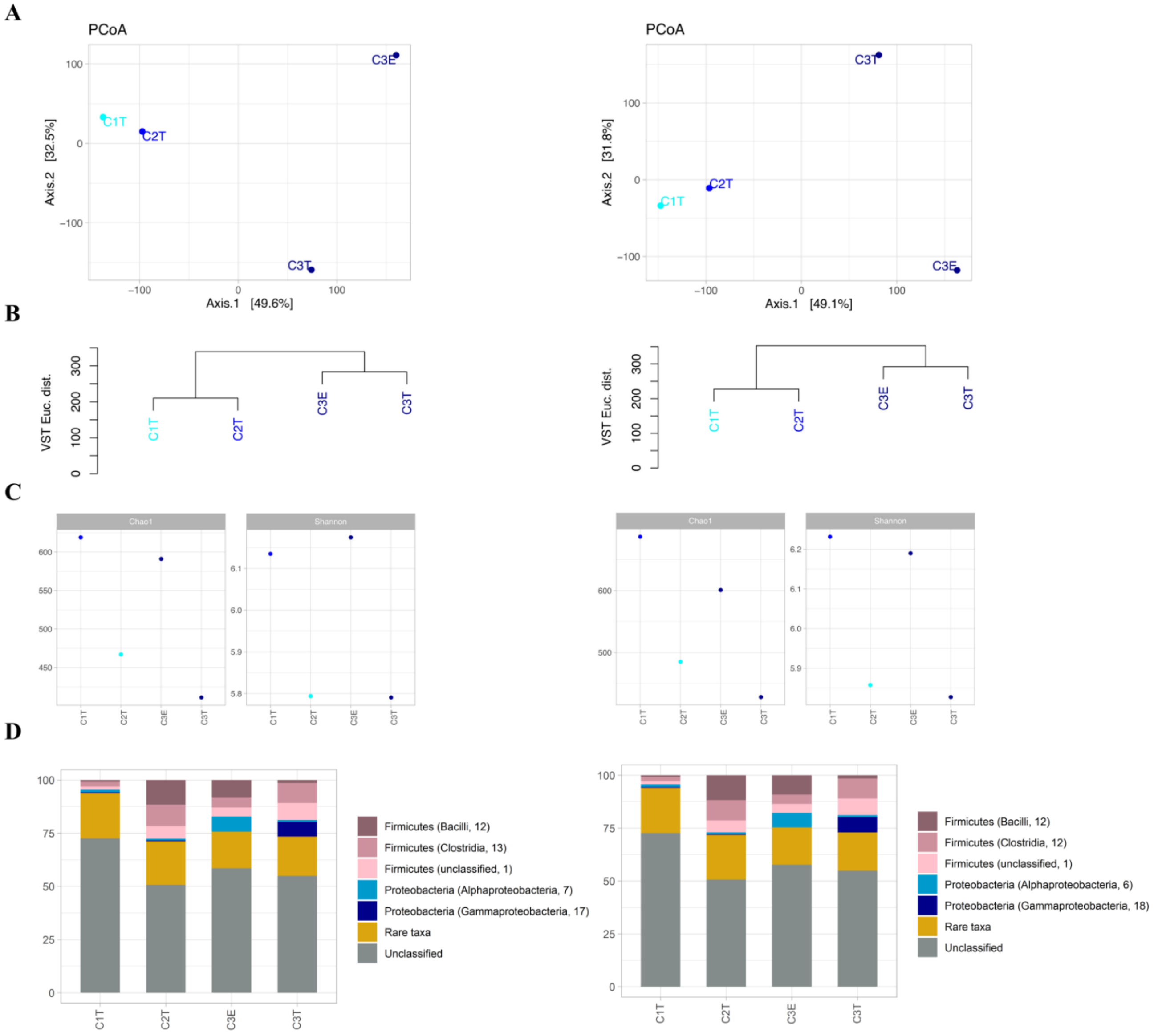

Figure S2. Trimming *in-silico* analysis (515F-806R dataset to 520F-785R length). To assess if sequencing reads length standardisation would lead to misleading biological interpretations, a 515F-806R subset (seagrass samples, n = 4) was manually trimmed to a 520F-785R length (a few bases from each end), using the *trimLeft = c(5,5)* and *trimRight = c(21,21)* parameters in DADA2. Clustering (**A**), ordination (**B**), alpha- (**C**) and beta-diversity (**D**) plots were compared between original (left) and trimmed (right) data. Taxonomic profiles at the class level are shown in D. Genera were collapsed into classes to avoid visual clutter and facilitate interpretation. Classes within each phylum are shown in brackets, with the number next to each class representing the number of genera. "Rare taxa" represent ASVs with relative abundance < 5% within each sample.

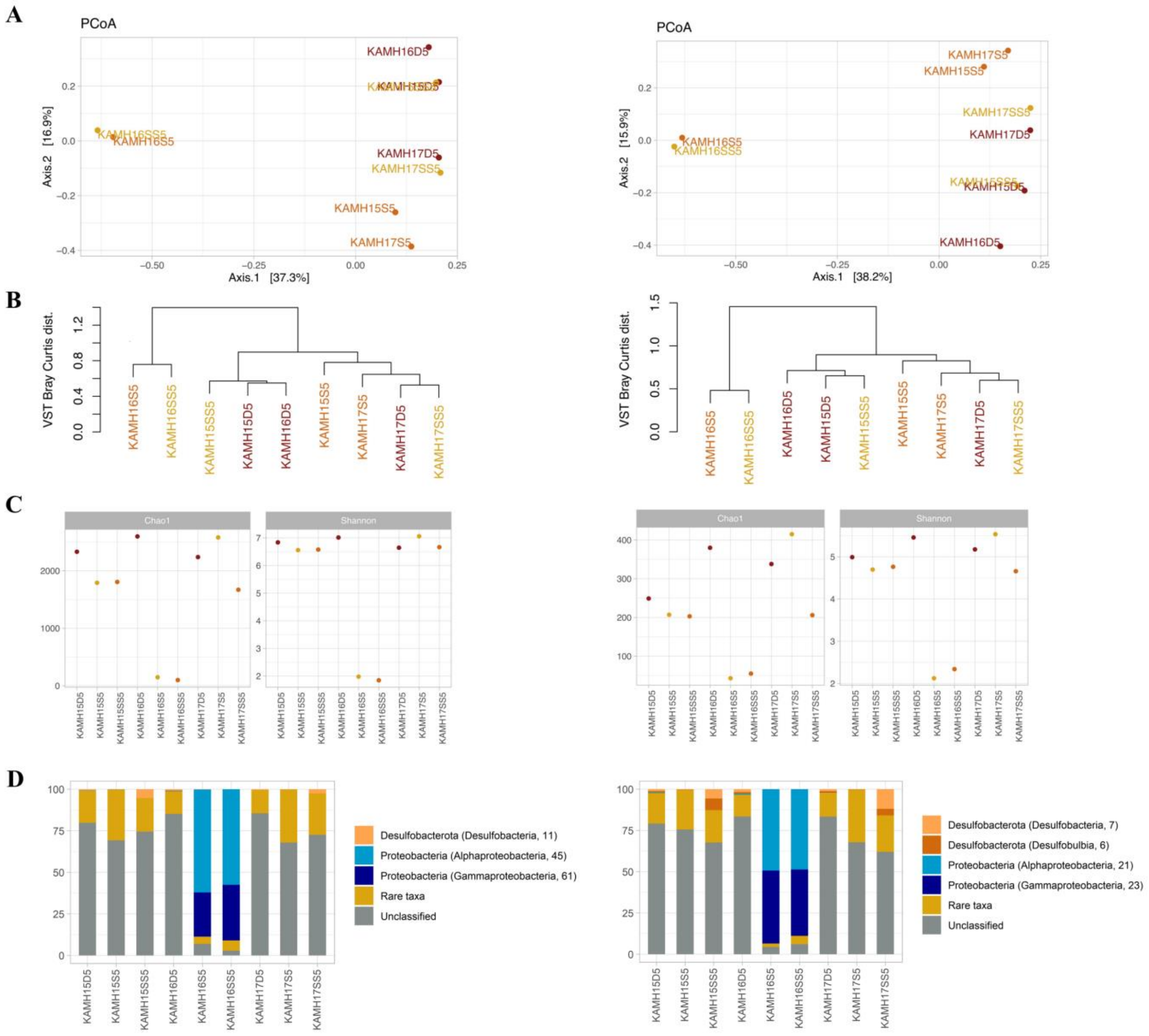

Figure S3. Trimming *in-silico* analysis (341F-785R dataset to 515F-785R length). To assess if sequencing reads length standardisation would lead to misleading biological interpretations, a 341F-785R subset (mangroves samples, n = 9) was trimmed to a 515F-785R length, using CUTADAPT as a treatment step before DADA2. Clustering (**A**), ordination (**B**), alpha- (**C**) and beta-diversity (**D**) plots were compared between original (left) and trimmed (right) data. Taxonomic profiles at the class level are shown in D. Genera were collapsed into classes to avoid visual clutter and facilitate interpretation. Classes within each phylum are shown in brackets, with the number next to each class representing the number of genera. “Rare taxa” represent ASVs with relative abundance < 5% within each sample.

2. Standardisation toolbox

2.1 Sequencing data

Standards for describing marker genes such as 16S rRNA genes were developed by the Genomic Standards Consortium (GSC) to capture the "minimum information" required to guide data integration, comparative studies, and ultimately knowledge generation [34]. Since its release more than a decade ago, several resources have been made available to aid submitting contextual data/metadata to most used data repositories (e.g., EBI-ENA and SRA) in compliance with GSC guidelines and its implementations – e.g., MetaBar [78]. Within this context, we could extrapolate ideas for metadata submission to other stages of the data acquisition process. Here, we propose standardisation tools for the acquisition of amplicon sequencing data.

2.1.1 Data collection

The minimum information about a marker gene sequence (MIMARKS) checklist was developed to include further experimental contextual data, such as PCR primers and the target gene, into nucleic acid and sequencing metadata [34]. This offered a great solution to the issue of lack of metadata without dealing with methods standardization. The resulting sufficient metadata from a wide range of sequencing approaches is inconvenient for comparative analyses. To tackle this issue of inconsistent metadata and aiming for consensus, we provide a set of preferred amplicon sequencing primers and platforms, based on the methods reported in the 34 studies compiled here and their use in global microbiome initiatives (**Table S1**). This list would help Blue Carbon soil microbiome researchers make well-informed decisions on sequencing approaches.

2.1.2 Data submission

There are several guidelines on requirements for the submission of sequencing data to publicly available repositories, which often comply with the minimum information about any (x) sequence (MIxS) specifications. Checklists and packages with "core metadata" are usually provided in the form of "electronic laboratory notebooks" to aid consistent reporting of marking gene investigations [34]. However, essential information such as PCR primer sequences or sample IDs that match the (usually shorter) labels used in publications are often missing. Moreover, sequencing reads are recurrently submitted in non-optimal formats. To solve these issues, submission processes would need to be modified. Taking MIMARKS as an example, we propose the modifications below to the existing checklist (**Table S2**). Other checklists for different types of data may need different adjustments.

- To include *target_subfragment*, *pcr_primers*, *mid*, and *adapters* into sequencing core items.
- To add *seq_type*, *data_format*, *read_length*, *data_status*, *sample_id_provider*, and *sample_id_author* to the checklist and sequencing core items.
- To enforce submission of core items.
- To rearrange position of items as they appear in the publication.
- To enable "export" option for all fields under sequencing core items.

2.1.3 Publication

The availability and accessibility of all data used in a submitted manuscript could be verified before it is accepted for publication by adding a data-check step to the peer-review process. This step would be similar to the reference check that takes place during the production process, but with emphasis on compliance with the minimum information about any (x) sequence (MIxS) specification.

2.2 Soil metadata and experimental designs

Similar levels of standardisation should be targeted for Blue Carbon metadata, specifically associated with soil biogeochemical parameters. There seems to be a range of resources to choose from when it comes to metadata management. However, the applicability of terrestrial soil methods to coastal or marine

environments is still uncertain, hence the high variability of approaches that we observed across BCEs microbiome studies. Here, we propose standardisation tools for the acquisition of Blue Carbon-related metadata for microbiome studies and approaches.

2.2.1 Data collection

Considering that several parameters can inform on Blue Carbon content, we first need to know what to measure and why. "Fine research" investigating cause-effect and correlative relationships between holobionts and the environments they occupy has been conducted for many years, elucidating general trends potentially relevant to Blue Carbon cycling and the role that microbes play in these processes. Our revision of BCE microbiome studies provides hypotheses of relevant standard Blue Carbon metrics. In line with recent discussions on Blue Carbon management strategies around soil biogeochemical parameters to establish organic carbon stocks – e.g., [79], our recommendation is to report concentration of carbon *and* depth interval: mass of carbon per unit area (mg C cm^{-2}) and the depth range, or mass of carbon per unit volume (mg C cm^{-3}) and the depth range when investigating the Blue Carbon soil microbiome.

2.2.2 Data format and accessibility

Secure metadata repositories like those available for genomic data are open to the scientific community with the purpose of archiving, publishing, and distributing high-quality data and metadata to advance the knowledge gained from synthesis research. Examples of these repositories include PANGAEA and the Environmental Data Initiative (EDI) data portal, both cited in our mined studies. PANGAEA is an information system focused on georeferenced data from earth system research (https://www.pangaea.de/), whereas the EDI data portal is a broader repository for environmental data in any digital format (https://environmentaldatainitiative.org/). While submission guidelines, platforms, and resources are accessible, and both organisations already hold thousands of datasets, there is not such a "culture of use" [80]. Consequently, summarised metadata from most of Blue Carbon studies is made available through figures and tables in published manuscripts, without the submission of the corresponding raw metadata to any of the available repositories. Taking EDI as an example, we propose an additional section to the existing metadata template (**File S3**), which is based on the machine readable Ecological Metadata Language – EML [81]. Namely, the "Blue Carbon metadata table" section, specifically targeted to seawater (or porewater, if feasible) physicochemical parameters and carbon content (i.e., mass levels) associated with rhizobiome or bulk soil samples that were also analysed through amplicon sequencing approaches. The suggested changes are consistent with metadata recording suggestions in the Blue Carbon Manual – see "Protocols" below [30]. We hope these changes would act as the equivalent of the minimum information about any (x) sequence (MIxS) specifications for Blue Carbon metadata, thus facilitating the standardization of the archiving process for further re-use. Please note that the proposed changes only apply for metadata associated with BCEs amplicon sequencing data.

2.2.3 Collaboration

The success of these strategies relies heavily on a collaborative approach, involving scientists from multiple disciplines who agree on standard methods for data collection, archival, and sharing. This will support further developments of platforms alike, ultimately facilitating future synthesis research. This paper is an invitation for everyone to contribute collaboratively to the creation of a culture of use.

2.3 Protocols

The Blue Carbon Manual was published as a part of the Blue Carbon Initiative in 2014 to standardise methods for measuring, assessing, and analysing carbon stocks and emissions factors in mangroves, tidal saltmarshes, and seagrass meadows [30]. This practical tool provides detailed protocols for sampling methods, laboratory measurements, and analysis of Blue Carbon stocks and fluxes, and can be used as the standard operational procedure (SOP) for collection, processing, and analysis of Blue Carbon soil microbiome samples. The manual included a Data Recording Worksheet for Soil Samples with guidelines on field and laboratory work. Based on this worksheet, dry bulk density and organic carbon content corrected for

inorganic portion are the two parameters that best inform on carbon mass levels. We propose a series of factors to keep constant formats and units, and the ideal ranges or limits for variable parameters (**File S4**). Ideal methods would allow for pairing microbiome samples to soil metadata in terms of depth and spatial resolution (i.e., using Blue Carbon data to help explain variability in microbiome structure and function). Alternatively, characterising Blue Carbon parameters at site-level (i.e., using Blue Carbon metadata to characterise overall site microbiome structure and to, more broadly, make comparisons across sites), if pairing is not feasible. This will provide scientists with reference values to coordinate global research and promote the production of robust, reusable Blue Carbon soil microbiome data. Different reference values may suit research interests other than comparative analyses between amplicon sequencing datasets. Therefore, standard methods and reference values may be shared with researchers around the world to encourage their application in the field. This would require further development of specialised shared data bases and platforms to expand on data entries related to carbon measurements in the rhizosphere. The ideal scenario, however, would be a single, centralised shared data base for established standard methods, protocols, and reference values. The Global Coastal Carbon Data Archive (https://www.thebluecarboninitiative.org/), the Coastal Carbon Research Coordination Network (CCRCN, https://serc.si.edu/coastalcarbon), and the Ocean Carbon and Acidification Data System (OCADS, https://www.ncei.noaa.gov/access/ocean-carbon-data-system/) are examples of local carbon data management systems currently available.

Table S1. Preferred sequencing approach. Preferred amplicon sequencing platforms (**A**) and primer sets (**B**) are shown in order of preference, based on a higher number of associated datasets and their reported use in large-scale projects or global initiatives.

A

Sequencing platform	Single vs. Paired –end reads	Read length (bp)	Associated data sets	Global initiatives
Illumina MiSeq	Paired – end	2 x 250	32	EMP, BASE, AusMic

B

Primer set	Sequence Fw (5'-3')	Sequence Rv (5'-3')	Associated data sets	Global initiatives
515F (Parada) – 806R (Apprill) versions	GTGYCAGCMGCCGCGGTAA	GGACTACNVGGGTWTCTAAT	16	EMP

Table S2. Modified MIMARKS checklist. Proposed changes to the original version of the checklist [34] are highlighted in grey fonts. Struc Com Name = structured comment name: name of a checklist item as it will appear in GenBank structured comments, MIMARKS survey and MIMARKS specimen = information about whether an item is mandatory (M), conditional mandatory (C), optional (X), environment-dependent (E) or not applicable (-) for a given checklist type, Occ = occurrence: indicates whether a given item can be used only once (1), multiple times (m), or none (0)), Pos = position: position of item as it appears in the publication. Seq = Sequencing.

Struc Com Name	Item	Definition	Example	Expected value	Section	MIMARKS survey	MIMARKS specimen	Value syntax	Occ	Pos
seq_meth	sequencing method	Sequencing method used; e.g., Sanger, pyrosequencing, ABI-solid	Sanger dideoxysequencing, pyrosequencing, polony	sequencing method	Seq	M	M	{text}	1	31
seq_type	sequencing type	Sequencing type used; e.g., single-read sequencing, paired-end sequencing	single-read sequencing, paired-end sequencing	sequencing type	Seq	M	M	{text}	1	32
target_gene	target gene	Targeted gene or locus name for marker gene studies	16S rRNA, 18S rRNA, nif, amoA, rpo	gene name	Seq	M	M	{text}	1	33
target_subfragment	target subfragment	Name of subfragment of a gene or locus. Important to e.g., identify special regions on marker genes like V6 on 16S rRNA	V6, V9, ITS	gene fragment name	Seq	M	M	{text}	1	34
pcr_primers	pcr primers	PCR primers that were used to amplify the sequence of the targeted gene, locus or subfragment. This field should contain all the primers used for a single PCR reaction if multiple forward or reverse primers are present in a single PCR reaction. The primer sequence should be reported in uppercase letters	-	FWD: forward primer sequence REV:reverse primer sequence	Seq	M	M	FWD:{dna} REV:{dna}	1	35

Struc Com Name	Item	Definition	Example	Expected value	Section	MIMARKS survey	MIMARKS specimen	Value syntax	Occ	Pos
mid	multiplex identifiers	Molecular barcodes, called Multiplex Identifiers (MIDs), that are used to specifically tag unique samples in a sequencing run. Sequence should be reported in uppercase letters	-	multiplex identifier sequence	Seq	M	M	{dna}	1	36
adapters	adapters	Adapters provide priming sequences for both amplification and sequencing of the sample-library fragments. Both adapters should be reported; in uppercase letters	-	adapter A and B sequence	Seq	M	M	{dna},{dna}	1	37
data_format	data format	Format of sequencing files	fasta, fastq	data format	Seq	M	M	{text}	1	38
read_length	read length	Read length for the sequencing run; i.e. the number of base pairs (bp) sequenced from a DNA fragment	250 bp, 300 bp	number of base pairs (bp)	Seq	M	M	{number}	1	39
data_status	data status	Processing level of data; i.e. how much of the analysis has been done to the raw reads by the sequencing provider or the author (s)	demultiplexed , joined, truncated	data status	Seq	M	M	{text}	1	40
sample_id_provider	sample id provider	Sample ID used by the sequencing provider	P6_cim_0101... P6_cim_0315 (from Moncada et al., 2019)	sample id provider	Seq	M	M	{text}	1	41
sample_id_author	sample id author	Sample ID used by the author (s)	S1_I_C1…S3_ PW_C5	sample id author	Seq	M	M	{text}	1	42
