# Supplementary material for: Pathways for Understanding Blue Carbon Microbiomes with Amplicon Sequencing
Source: Microorganisms. 2022 Oct 26;10(11):2121. doi: 10.3390/microorganisms10112121 (PMC9694552; doi:10.3390/microorganisms10112121)
Supplement: Supplementary file 1 [file microorganisms-10-02121-s001.zip › microorganisms-1921124_revised-sup-material_clean_proofread/5_microorganisms-1921124_files1_proofread.pdf]

**File S1.** PRISMA 2020 checklist.

| Section and Topic | Item # | Checklist item | Location where item is reported |
|---|---|---|---|
| **TITLE** | | | |
| Title | 1 | Pathways for Understanding Blue Carbon Microbiomes with Amplicon Sequencing | Main manuscript |
| **ABSTRACT** | | | |
| Abstract | 2 | The capacity of Blue Carbon Ecosystems to act as carbon sinks is strongly influenced by the me-tabolism of soil-associated microbes, which ultimately determine how much carbon is accumu-lated or returned to the atmosphere. The rapid evolution of sequencing technologies has facilitat-ed the generation of tremendous amounts of data on what taxa comprise below-ground microbial assemblages, largely available as isolated datasets, offering an opportunity for synthesis research that informs progress on understanding Blue Carbon microbiomes. We identified questions that can be addressed with a synthesis approach, including the high variability across datasets, space, and time due to differing sampling techniques, ecosystem or vegetation specificity, and the rela-tionship between microbiome community and edaphic properties, particularly soil carbon. To address these questions, we collated 34 16S rRNA amplicon sequencing datasets, including bulk soil or rhizosphere from seagrass, mangroves, and saltmarshes within publicly available reposi-tories. We identified technical and theoretical challenges that precluded a synthesis of multiple studies with currently available data, and opportunities for addressing the knowledge gaps within Blue Carbon microbial ecology going forward. Here, we provide a standardisation toolbox that supports enacting tasks for the acquisition, management, and integration of Blue Carbon-associated sequencing data and metadata to potentially elucidate novel mechanisms be-hind Blue Carbon dynamics. | Main manuscript |
| **INTRODUCTION** | | | |
| Rationale | 3 | A standard methodology to tease apart the driving forces of Blue Carbon microbiomes structure, their role in global carbon cycling processes, and their interaction with organic carbon and the environment across multiple interacting levels (i.e., compartments, organisms, and habitats) is currently lacking. Nevertheless, below-ground microbiomes associated with Blue Carbon Ecosystems (BCEs) have been studied separately through amplicon sequencing approaches, delivering theoretically suitable datasets for meta-analysis – a very powerful predictive tool that would extend our understanding of coastal and marine microbiomes, while facilitating the generation of more targeted hypotheses for future research. A meta-analysis approach may be a valuable way to utilise already existing datasets to produce novel insights into BCEs microbiomes beyond the scope of a single study. Such meta-analysis can offer critical insights into the current state of knowledge on soil microbiomes associated with BCEs, while elucidating subtle but informative patterns from complex data. Our approach would advance our understanding of below-ground microbiome dynamics in BCEs and generate potentially valuable knowledge for the development of new microbiome methodologies. Moreover, generalised compositional changes (or absence of them) in Blue Carbon below-ground microbiomes could be used to establish microbial baselines to assess disturbance effects, and microbial predictors of carbon sequestration and soil health. | Main manuscript |
| Objectives | 4 | Our objective was to use a meta-analysis approach to address five key knowledge gaps that are relevant to the small (within an ecosystem or soil core) and large (between systems or transition zones) scales. These interrogate whether | Main |

**File S1.** PRISMA 2020 checklist.

| Section and Topic | Item # | Checklist item | Location where item is reported |
|---|---|---|---|
| | | Blue Carbon ecosystems have a distinct soil microbiome, inter- and intra-specific and scale-based variation in their soil microbiomes, and the possible role of soil carbon density and other environmental forcing in shaping Blue Carbon soil microbiomes:<br>• Is there a Blue Carbon soil microbiome or a shared "Blue Carbon microbial signature" between BCEs?<br>• Is the Blue Carbon microbiome linked to soil carbon content and other Blue Carbon soil metrics?<br>• What is the effect of other environmental and edaphic parameters on the Blue Carbon soil microbiome?<br>• Do inter- and intra-specific variation influence soil microbiomes in BCEs?<br>• Would this Blue Carbon signature change across different spatio-temporal scales? | manuscript |
| **METHODS** | | | |
| Eligibility criteria | 5 | **Data sets:** we collated data derived from amplicon (16S rRNA gene) sequencing of microbial assemblages associated with seagrasses, mangroves, and saltmarshes to explore patterns in Blue Carbon microbiomes.<br>**Sample types:** we considered both the rhizosphere (i.e., microbes closely associated with the roots/rhizomes) and bulk soil microbiome (i.e., microbes associated with bare soils) while reviewing the literature to account for the within- and between-ecosystem scales at which the Blue Carbon soil microbiome is studied.<br>**Studies inclusion:** we included studies with publicly available raw 16S rRNA data (read files in FASTQ or FASTA formats), sequenced using the Illumina platforms MiSeq or HiSeq (paired-end reads) with primers targeting the V3-V4 hypervariable regions of the 16s rRNA gene (e.g., 515F/806R), and with metadata indicating the type of sample (rhizosphere vs. bulk soil), soil biogeochemistry (e.g., bulk density, grain size, texture, etc.) or carbon/nitrogen content (e.g., total C, % organic C, dissolved organic C, C:N ratio, etc.). | Main manuscript, Supplementary Materials |
| Information sources | 6 | We first surveyed the literature to identify microbiome studies on BCEs, using key-word searches in the Google Scholar, Scopus, and Web of Science databases (June – December 2021). Additional studies were identified by following references in related microbiome studies on coastal, marine, and estuarine soils not specifically associated with the rhizosphere. Sequencing reads and corresponding metadata were downloaded from online repositories – e.g., read files from Sequence Read Archive (SRA) or European Nucleotide Archive (ENA), metadata from PANGAEA or Environmental Data Initiative (EDI), or links provided in the original publications, or were acquired directly from the authors. More details of the information sources in File S2. | Supplementary Materials, File S2 |
| Search strategy | 7 | Manual key-word searches in the Google Scholar, Scopus, and Web of Science databases included the following terms: "16S", "rRNA", "microbiome", "microbial", "seagrass", "mangrove", "saltmarsh", "salt marsh", "tidal marsh", "wetland", "sediment", "rhizosphere", "carbon", "nutrient", "Illumina", and "MiSeq". A multi-faceted, peer-reviewed approach was used, whereby a series of searches were performed with different terminology combinations to refine keywords with synonyms. Additionally, reference cited in microbiome studies on coastal, marine, and estuarine soils that were not specifically associated with the rhizosphere were manually examined for the same terminology. | Supplementary Materials |

| Section and Topic | Item # | Checklist item | Location where item is reported |
|---|---|---|---|
| | | **Search strategy development process:** six known relevant microbiome studies (two per habitat type) were used to identify related records within databases. Keywords were identified by looking at microbiome terminology in the titles and abstracts of these records and combined into a single search line that was used as a search strategy. The search strategy was validated by testing that it could identify at least the six known relevant studies in all databases. The strategy was internally developed, and the search outcomes (collated data) were initially peer-reviewed by two experienced biogeochemist and microbial ecologists at two different stages of the project. Peer review initially involved proofreading the syntax, spelling, and overall study structure, as well as verification of inclusion decisions for sample types. | |
| Selection process | 8 | Two researchers independently reviewed titles and abstracts of all records divided in two groups in accordance with their expertise: Ashley Bulseco screened records associated with saltmarshes, while VHM screened records associated with seagrass and mangroves. The double screening was then completed by cross-checking titles and abstracts of all screened records. Next, full-text articles were screened using the same approach (combined single and double reviewer assessments), with emphasis on the methods of pre-screened studies. A third, independent reviewer checking of all records was needed to resolve disagreements between screeners and reach consensus on not specified parameters. Screening decisions for manually checked records were used for subsequent data subsetting steps to avoid unnecessary reassessment. | N/A |
| Data collection process | 9 | A data extraction spreadsheet was used to mine data from eligible studies divided in the same two expertise groups (see item 8). This process was partially verified by a third reviewer (STT cross-checked sample types). Disagreements between data collectors were discussed until consensus was reached. Although not always successful, email communications with authors and journal editors were used to obtain relevant data not included in final publications (e.g., links to raw sequencing files or metadata) or to clarify any missing or unclear information. Data collection was not finalised due to unsuitability of the data for meta-analysis. | N/A |
| Data items | 10a | We did not get to refining data items into more specific results within each outcome domain due to inconsistencies/incompatibilities across data sets, but the following data were preliminarily sought:<br>Sampling technique – type of habitat sampled (vegetated, unvegetated or vegetated-unvegetated).<br>Sequencing method – sequencing platform and primer set used.<br>Experimental design – assessing the effect of environmental condition, host species or phenotype, microenvironment, depth, impact, or time.<br>Soil metadata – recorded physicochemical (in seawater or sediment), oxygen level, bulk density, edaphic, carbon level, nitrogen level, carbon to nitrogen ratio, trace metals, inorganic nutrients, pore water nutrients, or other parameters.<br>All results that were compatible with each outcome domain in each study were initially sought (all bulk soil and rhizosphere samples within each study), with the intention to later selecting the outcome definition that was most | Main manuscript, File S2 |

| Section and Topic | Item # | Checklist item | Location where item is reported |
|---|---|---|---|
| | | common across studies and decide which results to collect. | |
| | 10b | **Variables of interest:** additional variables of interest included location (city and country), sample type (bulk soil, rhizosphere, roots), data source (data bases and repositories), and target microorganism (bacteria, archaea, microalgae, fungi).<br>**Assumptions:** sampling depth for bulk soil samples was inferred from described methodology in the manuscripts if not explicitly stated, unclear, or not confirmed by the authors. When "surficial" samples were reported, the sampling depth was assumed to be 0 – 1 cm. | File S2 |
| Study risk of bias assessment | 11 | Risk of bias was not assessed because the meta-analysis with the current available data was deemed not possible after preliminary *in- silico* analyses. These analyses showed that differences in read length (i.e., short amplicons not covering the desired region), composition (i.e., absence of primers in the reverse reads), and pre-processing prior to database submission in some of the sequencing data prevented trimming all reads to the same length, and subsequently pooling of these datasets. Consequently, this meta-analysis did not progress to the data extraction and syntheses stages. | N/A |
| Effect measures | 12 | Data extraction was not performed (refer to item 11 for details). | N/A |
| Synthesis methods | 13a | | N/A |
| | 13b | | N/A |
| | 13c | | N/A |
| | 13d | | N/A |
| | 13e | | N/A |
| | 13f | | N/A |
| Reporting bias assessment | 14 | | N/A |
| Certainty assessment | 15 | | N/A |
| **RESULTS** | | | |
| Study selection | 16a | We found 130 records in databases searching. After 99 duplicates were removed, we manually screened 31 records, from which 3 records were excluded because of unsuitable experimental designs. We also searched documents that cited any of the initially included studies as well as the references of the initially included studies, and 7 extra articles that fulfilled the inclusion criteria were found in these searches. An additional record was excluded because of its small sample size. A total of 34 full-text documents were assessed for eligibility. However, our intended meta-analysis was declared unsuitable because of issues with the sequencing data and meta-data. At this stage, no further records | Figure S1 |

File S1. PRISMA 2020 checklist.

| Section and Topic | Item # | Checklist item | Location where item is reported |
|---|---|---|---|
| | | were screened or excluded. Please refer to the flow diagram provided along with this checklist. | |
| | 16b | The studies below appeared to meet the inclusion criteria but were excluded for the following reasons:<br>- Records excluded due to unsuitable design (e.g., manipulative laboratory experiments):<br>Martin et al. (2018): https://doi.org/10.3389/fmicb.2017.02667<br>Trevathan-Tackett et al. (2017): https://doi.org/10.1093/femsec/fix033<br>Moncada et al. (2019): https://doi.org/10.1093/femsec/fiz006<br>- Reports excluded due to small sample size:<br>Leadbeater et al. (2021): https://doi.org/10.1186/s40168-020-00964-0<br><br>No reports were excluded after assessing for eligibility, but the meta-analysis was declared unsuitable for the following reasons:<br>- Incomplete raw sequencing data:<br>Marcos et al. (2018): https://doi.org/10.1007/s00248-017-1091-y<br>Booth et al. (2019): https://doi.org/10.1038/s41598-019-40315-0<br><br>- Other sequencing data formatting issues:<br>Zhou et al. (2017): https://doi.org/10.3389/fmicb.2017.02148<br>Torres et al. (2019): https://doi.org/10.1016/j.envpol.2019.113293<br>Booth et al. (2019): https://doi.org/10.1038/s41598-019-40315-0<br>Booth et al. (2019): https://doi.org/10.1038/s41598-019-43980-3<br>Liu et al. (2018): https://doi.org/10.1002/mbo3.600<br>Cúcio et al. (2016): https://doi.org/10.3389/fmicb.2016.00440<br><br>- Unavailable soil metadata:<br>Fahimipour et al (2017): https://journals.asm.org/doi/epub/10.1128/AEM.03391-16<br>Hurtado-McCormick et al. (2019): https://doi.org/10.3389/fmicb.2019.01011<br>Kolton et al. (2020): https://doi.org/10.1093/femsec/fiaa026 | |
| Study characteristics | 17 | Study characteristics are summarised in File S2. | File S2 |
| Risk of bias in studies | 18 | Syntheses were not conducted (refer to item 11 for details). | N/A |
| Results of individual studies | 19 | | N/A |
| Results of syntheses | 20a | | N/A |

**File S1.** PRISMA 2020 checklist.

| Section and Topic | Item # | Checklist item | Location where item is reported |
|---|---|---|---|
| | 20b | | N/A |
| | 20c | | N/A |
| | 20d | | N/A |
| Reporting biases | 21 | | N/A |
| Certainty of evidence | 22 | | N/A |
| **DISCUSSION** | | | |
| Discussion | 23a | | N/A |
| | 23b | | N/A |
| | 23c | | N/A |
| | 23d | | N/A |
| **OTHER INFORMATION** | | | |
| Registration and protocol | 24a | This systematic review was not registered. | N/A |
| | 24b | A review protocol was not prepared. | N/A |
| | 24c | N/A | N/A |
| Support | 25 | **Funding**: this project was supported by an Australian Research Council (ARC) Discovery Project (DP200100575). STT was supported by an ARC Discovery Early Career Researcher Award (DECRA) Fellowship (DE210101029).<br>**Role of funder:** the funders had no role in the design of the systematic review, data collection and analysis, decision to publish, or preparation of the manuscript. | Main manuscript |
| Competing interests | 26 | **Declarations of interests:** Ashley Bulseco, VHM, and STT are authors of the following reports reviewed to collate the data:<br>Ashley Bulseco was author of Kearns et al. (2019) and Lynum et al. (2020).<br>VHM was first author of Hurtado-McCormick et al. (2019).<br>STT was first author of Trevathan-Tackett et al. (2020). | N/A |
| Availability of data, code and other materials | 27 | All data and other materials relevant to our intended systematic review were described in detail in the scientific publication submitted as "Challenges and opportunities of a blue carbon microbiome meta-analysis" to Microorganisms. Additional information to reuse the data, check the data for errors, attempt to reproduce the findings, or understand more about our intended meta-analysis may be available upon request. Contact: Valentina Hurtado-McCormick, v.hurtadomccormick@deakin.edu.au | Main manuscript, Supplementary Materials |

**File S1.** PRISMA 2020 checklist.

Adapted from http://www.prisma-statement.org/ [77].
