# Supplementary material for: Pathways for Understanding Blue Carbon Microbiomes with Amplicon Sequencing
Source: Microorganisms. 2022 Oct 26;10(11):2121. doi: 10.3390/microorganisms10112121 (PMC9694552; doi:10.3390/microorganisms10112121)
Supplement: Supplementary file 1 [file microorganisms-10-02121-s001.zip › microorganisms-1921124_revised-sup-material_clean_proofread/7_microorganisms-1921124_files3_proofread.pdf]

File S3. Suggested additional section for Blue Carbon associated metadata recording. Modifications to the existing "data table" section in the metadata template from the Environmental Data Initiative (EDI) data portal are consistent with metadata recording suggestions in the Blue Carbon Manual [30].

Source: https://environmentaldatainitiative.org/submit-your-data/

Blue carbon metadata table

Attach this table to each raw metadata table submitted to the portal, indicating any associated graphical summaries (figures) or average values (tables).

Provide a "table name" and "table description".

Each row in the below table describes one column in your data table.

Complete each row as follows:

- ***Column name**: This name must be exactly as it appears in the dataset. Please avoid special characters (like & or \), dashes and spaces. Underscores are permissible. Do not begin a column name with a number.*

- ***Description**: Please give a specific definition of the column name. This can be lengthy.*

- ***Unit:** Identify units for all numeric variables. Please avoid special characters and describe units in this pattern: e.g. microSiemenPerCentimeter, microgramsPerLiter, absorptionPerMolePerCentimeter.*

- ***Code explanation**: If you use codes in your column, please explain in this way: e.g., LR=Little Rock Lake, A=Sample suspect, J=Nonstandard routine followed.*

- ***Date format**: Please tell us exactly how the date and time is formatted: e.g. dd/mm/yyyy hh:mm:ss plus the time zone and whether or not daylight savings was observed. ISO date format of YYYY-MM-DD or YYYY-MM-DD hh:mm:ss is preferred.*

- ***Missing value code**: If a code for 'no data' is used, please specify: e.g., -99999.*

Table name: Blue Carbon associated metadata

Table description: water physicochemical parameters and carbon mass levels associated with rhizobiome samples that were also analysed through amplicon sequencing approaches.

Associated graphical summaries (figures): Figure x, y, and z in {reference}.

Associated average values (tables): Table a, b, and c in {reference}.

Column name	Description	Unit / Code explanation / Date format	Missing value code
Water temperature	Temperature of the water column	degreeCelsius / nm	Blank / nm = not measured
Water pH	pH of the water column	None / nm	Blank / nm = not measured
Water salinity	Salinity of the water column	practicalSalinityUnit / nd	Blank / nd = not detected
Water conductivity	Conductivity of the water column	microSiemenPerCentimeter / nd	Blank / nd = not detected
Dry bulk density	Sediment mass (weight) of the dry solids divided by the total volume of the wet sample	gramPerCubicCentimetre / nm	Blank / nm = not measured
Inorganic carbon content	Inorganic carbon portion of the sediment sample, determined by acidification methods or elemental analysis	%	Blank / nm = not measured
Organic carbon content	Organic matter stock of the sediment sample, corrected for inorganic portion	%	Blank / nm = not measured
