# Supplementary material for: Pathways for Understanding Blue Carbon Microbiomes with Amplicon Sequencing
Source: Microorganisms. 2022 Oct 26;10(11):2121. doi: 10.3390/microorganisms10112121 (PMC9694552; doi:10.3390/microorganisms10112121)
Supplement: Supplementary file 1 [file microorganisms-10-02121-s001.zip › microorganisms-1921124_revised-sup-material_clean_proofread/8_microorganisms-1921124_files4_proofread.pdf]

File S4. Suggested reference values for data collection. Factors relevant to the standard collection of sediment samples for amplicon sequencing approaches and subsequent meta-analysis, with corresponding values. Data formats, measurement units, and proposed values are provided each parameter listed in the "data recording worksheet for soil samples" – Appendix G in the Blue Carbon Manual [30].

Appendix G – Data recording worksheet for soil samples

1. Field work

Person/Institution (and contact information)
{text} = any

Date
{dd-mm-yyyy} = any

Hour and tide information
{hh-mm-ss} = any
{text} = any

Core ID
{text/value} = any

General location (area, country)
{text, text} = any

GPS position
{ddd° mm′ ss.s″} = any

Depth of water column (if applicable)
{value} = any

Coring device material
{text} = any

Internal diameter of the core (cm)
{value} = at least x

Total length of the corer (cm)
{value} = a-b (seagrass), c-d (mangroves), e-f (salt marshes)

Coring system
{text} = any

Corer-end (cutting head/syringe)
{text} = cutting head/syringe

Coring vertically (Y/N)?
{text} = Y

Total length of corer outside sediment after core insertion (cm)
{value} = less than x

Total length of soil core (cm) – paired-design *
{value} = g-h (seagrass), i-j (mangroves), k-l (salt marshes)

Sliced in X cm-slices (whole core or hemi-core?) – paired-design *
{value} = m, whole/hemi (seagrass); n, whole/hemi (mangroves); o, whole/hemi (salt marshes)

Total number of samples – paired-design *
{value} = at least 3 per sample type

**** Paired-design:***
- Ideally, these parameters should match the depths of microbiome sampling.
- Alternatively, depth ranges should encompass the microbiome depths sampled.
- A minimum of 3 replicates (ideally >5) for a site-level characterisation.

Notes/comments

E.g., Coring issues? Sealing correct? Study site: Plant density/cover? Additional pictures of sampling site? Presence of shells, gravel, mud, plant debris, etc.

Visual description of the core (high-resolution digital picture)

- *Bulk sediment samples should be collected as specified above.*
- *Rhizosphere samples (sediment detached from roots/rhizomes by shaking) should collected at the same locations.*
- *Habitat-feature data and water physicochemical parameters (pH, salinity, electrical conductivity, temperature) should be recorded on-site.*

2. Lab – Create a sheet for each sample

Person/Institution
{text} = any

Date
{dd-mm-yyyy} = any

Core ID
{text/value} = any

Sample ID
{text/value} = any

Slice depth (cm) – paired-design *
{value} = a-b (seagrass), c-d (mangroves), e-f (salt marshes)

Slice thickness (cm) – paired-design *
{value} = g-h (seagrass), i-j (mangroves), k-l (salt marshes)

Dry bulk density (g/cm^3)
{value} = any

Carbonate present (Y/N)?
{text} = any

Method used to determine inorganic carbon content
{text} = acidification methods/elemental analysis

Inorganic carbon content (%)
{value} = any

Organic carbon content (corrected for inorganic portion, g)
{value} = any

** **Paired-design:***
- Ideally, these parameters should match the depths of microbiome sampling.
- Alternatively, depth ranges should encompass the microbiome depths sampled.
- A minimum of 3 replicates (ideally >5) for a site-level characterisation.

Notes/comments

E.g., Any deviations from standard operating protocols? Any machinery malfunctions?
Standard operating protocols from this manual should be used to determine carbon content.

3. Lab – Create a sheet for each core

Person/Institution
{text} = any

Date
{dd-mm-yyyy} = any

Core ID
{text/value} = any

Corresponding sample IDs
{text/value} = any

Total carbon in core (MgC)
{value} = any

FINAL CARBON ANALYSIS

Person/Institution
{text} = any

Date
{dd-mm-yyyy} = any

Location
{text} = any

Number of cores taken – paired-design *
{value} = at least x

Average carbon content of the cores (MgC)
{value} = any

Total area of strata (m^2)
{value} = at least x

Total soil carbon (per top X cm) of the strata (MgC/ha, in top X meters of soil) – paired design *
{value} = any, in top x centimeters of soil

*** Paired-design:**
- Ideally, these parameters should match the depths of microbiome sampling.
- Alternatively, averages to represent site overall (≥ 3 samples per site).
